# Alkylative Ring-Opening of Bicyclic Aziridinium Ion and Its Application for Alkaloid Synthesis

**DOI:** 10.3389/fchem.2019.00460

**Published:** 2019-06-27

**Authors:** Nagendra Nath Yadav, Young-Gun Lee, Nikhil Srivastava, Hyun-Joon Ha

**Affiliations:** ^1^Department of Chemistry, North Eastern Regional Institute of Science and Technology, Nirjuli, India; ^2^Department of Chemistry, Hankuk University of Foreign Studies, Yongin, South Korea

**Keywords:** aziridine, bicyclic aziridinium ions, alkylation, ring-expansion, alkaloid synthesis

## Abstract

Alkylative ring-opening of bicyclic aziridinium ion generated from 4-hydroxybutylaziridine with organocopper reagent was achieved successfully to afford 2-alkylsubstituted piperidine in high or moderate yield. This method allowed carbon-carbon bond formation of “non-activated” aziridine via aziridinium ion ring-opening in regio- and stereo-selective manner for the first time. This newly developed reaction was applied for an efficient synthesis of alkaloid with the representative example of conine and epiquinamide.

**Graphical Abstract:**
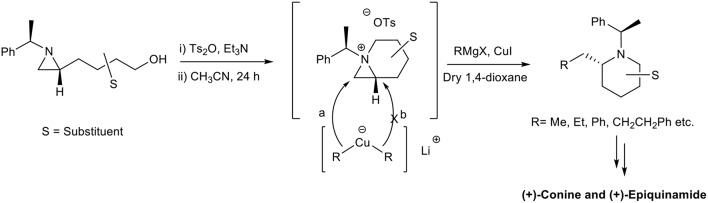
Alkylative ring-opening of bicyclic aziridinium ion for alkaloid synthesis.

## Introduction

Aziridine as a nitrogen-containing three membered ring has high ring-strain described as a “spring-loaded” compound by Yudin in his book (Yudin, 2006). This ring strain has two different aspects as unstability and high reactivity. Comparing to other popular three membered rings such as cyclopropane and oxirane (epoxide), aziridine has an extra-commander to control of two different aspects by the change of the characteristics of aziridine ring-nitrogen (Yudin, 2006). When the aziridine ring-nitrogen has electron-attracting substituent we call it “activated aziridine” with instability and high activity. Most of these aziridines are quite reactive toward the aziridine-ring opening with the most nucleophiles including carbon nucleophiles (Ghorai et al., 2011). However, so called “non-activated aziridine” (D'hooghe and Ha, 2016) with strong basic ring nitrogen having electron-donating substituents such as alkyl groups are not reactive toward either common alkyl nucleophiles or quite reactive heteroatom nucleophiles such as azide, acetate, alkoxide. For the successful reactions with these heteroatom nucleophiles, the aziridine ring should be activated as aziridinium ion in the presence of acids (Kim et al., 2006; Stanković et al., 2012). In past few years we succeeded in generation of alkyl aziridinium ions as their stable forms in inter- and intramolecular manner and their successful ring opening with various heteroatom nucleophiles to give multi-substituted nitrogen-containing valuables in high regio- and stereo-specific manner (Kim et al., 2008; Dolfen et al., 2016; Boydas et al., 2018). However, limited number of alkylative aziridine-ring opening was succeed only after N-methylation by highly reactive methyl triflate (Yoon et al., 2012).

In this communication we would like to report generation of 1-azoniabicyclo[4.1.0]heptanes tosylate as one of aziridinium ions and its regio- and stereo-specific ring opening for the synthesis of chain extended 2-alkylpiperidine. This chemistry has been used for efficient synthesis of alkaloids such as conine and epiquinamide as representative examples (Graphical Abstract). Efficient and stereoselective construction of aza-heterocycles have always been a challenge for synthetic organic chemists because they played a very important role in the field of pharmaceutical industry (Bailey et al., 1998; Husson and Royer, 1999; Passarella et al., 2002; Carry et al., 2013; Yadav et al., 2016). Among them piperidines as a six-membered aza-heterocycles are present in large number of biologically active natural products especially in the class of alkaloids. Many novel synthetic approaches have been developed for aza-heterocycles in an efficient manner to encounter regio-and stereoselective demand for the drug specification (Nicolaou et al., 1995; Masse et al., 2000; Kumar and Bodas, 2005; Trost et al., 2007; Srivastava and Panda, 2008; Chavan et al., 2015). However, no synthetic method is available to construct piperidine ring with concomitant introduction of the proper alkyl group at the alpha-position of the piperidine ring. Recently, we succeeded in regio- and stereoselective ring opening reaction of stable bicyclic aziridinium ion by diverse heteroatom nucleophiles to get various pipiridines and azepane in good to excellent yields (Ji et al., 2014; Eum et al., 2015; Dolfen et al., 2016; Choi et al., 2017; Yadav and Ha, 2018; Macha et al., 2019). In continuation of this chemistry, we herein report the alkylative, regio-, and stereo-selective ring-opening of 1-azoniabicyclo[4.1.0]heptanes tosylate by various organocopper reagents to afford piperidines with carbon-chain extension at C2 position. This selective transformation allow us to access various biologically important natural products with representative examples including (+)-conine (Hattori and Yamamoto, 1993; Hirai and Nagatsu, 1994; Munchhof and Meyers, 1995; Reding and Buchwald, 1998), and (+)-epiquinamide (Suyama and Gerwick, 2006; Tong and Barker, 2006; Srivastava et al., 2009; Airiau et al., 2010) in highly efficient manner.

## Results and Discussion

**Scheme 1:**
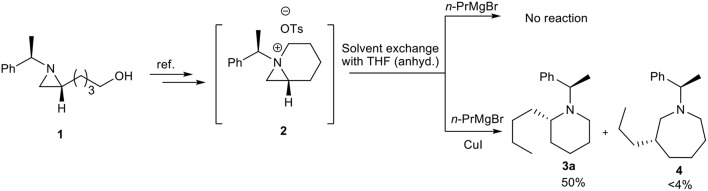
Regioselective synthesis of 2-alkyl substituted piperidine.

The bioavailability and importance of 2-alkyl piperidines as key intermediate for synthesis of various bioactive compounds encourage us to develop an efficient method for these systems from chiral aziridines. Our recent report on formation of stable 1-azoniabicyclo[4.1.0]heptanes tosylate **2** and its subsequent ring opening by the heteroatom nucleophile to give heteroatom-substituted piperidine and azepanes, promoted us to explore carbon-chain extension with concomitant highly strained aziridine-ring expansion with carbon nucleophile. Initially, we have treated freshly prepared bicyclic aziridinium ion **2** with *n*-PrMgBr in dry THF but the desired ring expanded product **3** was not formed. This may be due to hardness of the nucleophile generated from Grignard reagent (Kozlowski, 1991). Then we added CuI as reagent for *in situ* generation of organocopper reagents, which can changed the nature of nucleophile (Kozlowski, 1991) and we obtained the required product in 50% yields (Scheme 1). Furthermore, the reaction is highly regioselective and ring-opening proceeded by nucleophilic attack at C2 of aziridinium ion **2** to give piperidine **3a** along with formation of traces of azepane compound **4** by nucleophilic attack at C3.

Once we isolated and characterized compound **3a**, we tried to improve the regioselectivity and yields. We have screened several different Cu(I) salts and solvents to carry the reaction. We found that the reaction of bicyclic aziridinium tosylate **2** with *n*-PrMgBr in anhydrous 1,4-dioxane in the presence of CuI gave the desired product **3a** in 70% yields (Table 1).

**Table 1:** Optimization of reaction conditions for ring expansion of aziridinium ion with *in situ* generated organocopper reagents.

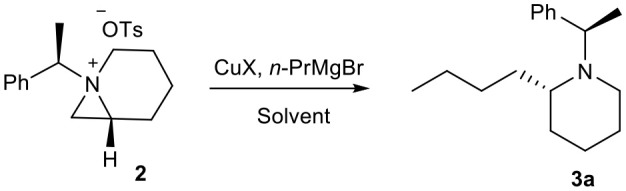					
**Entry^a^**	**Reagent^b^**	**Solvent**	**Temp. (°C)**	**Time (h)**	**Yield (%)^c^**
a	CuCN	THF	0–25	1.0	15
b	CuCN	1,4-dioxane	0–25	1.0	24
c	CuBr	THF	0–25	1.0	20
d	CuI	1,4-dioxane	0–25	1.0	70
e	CuBr	1,4-dioxane	0–25	1.0	32
f	CuI	THF	0–25	1.0	50

a *Reaction was performed at 0.5 mmol*.

b *Copper reagent 1.5 equiv., Grignard 3.0 equiv*.

c *Yield refers to pure products*.

Once we have optimized reaction condition for alkylative ring opening of aziridinium ion by organocopper reagents in hand, we used various reagents generated *in situ* from Grignard reagents and CuI in 1,4-dioxane to get various piperidine **3a**–**l** as shown in Table 2. Reagents having non-hindered alkyl group such as *n*-propyl **3a**, methyl **3f**, ethyl **3b**, and allyl **3e** resulted into the expected product in good yields (>50%), while those having hindered and bulky alkyl groups such as benzyl **3c**, *n*-nonyl **3d**, phenyl **3g**, *iso*-propyl **3i**, *p*-chlorophenyl **3k**, and cyclopentyl **3l** gave poor yields (25–38%) due to possible hindrance for the alkyl nucleophiles to approach the aziridinium ions.

**Table 2:**
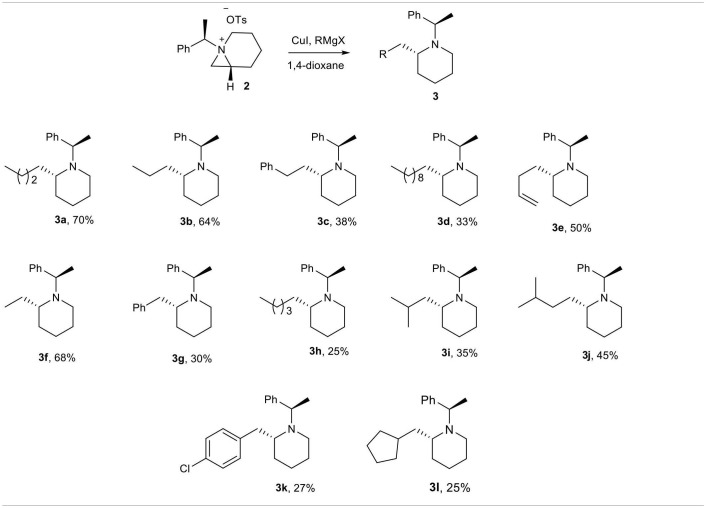
Synthesis of 2-alkyl piperidines from aziridine.

**Figure 1:**
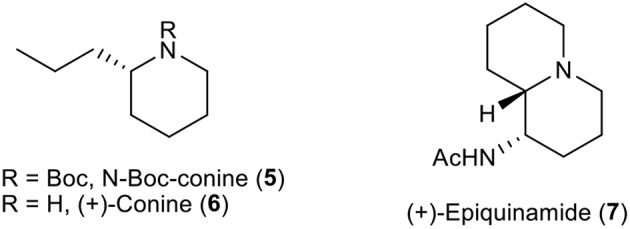
Conine **6**, its Boc-protected form **5** and Epiquinamide **7**.

After successful development of a new method to prepare 2-alkyl substituted piperidines from chiral aziridines, our attention was to apply this reaction for a short and efficient synthetic route for few biologically active molecules having piperidine skeleton such as (+)-conine **6** and (+)-epiquinamide **7** (Figure 1).

**Scheme 2:**
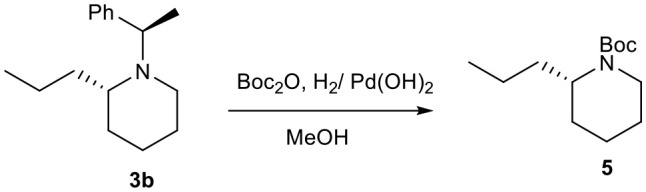
Synthesis of *N*-Boc-conine **5**.

Optically active conine, the poisonous hemlock alkaloid is a very important among piperidines alkaloids (Hattori and Yamamoto, 1993; Hirai and Nagatsu, 1994; Munchhof and Meyers, 1995; Reding and Buchwald, 1998; Jo et al., 1999; Pachamuthu and Vankar, 2001; Chacko and Ramapanicker, 2015). Using our strategy, we were able to synthesized *N*-Boc-conine **5** in optically pure form in few steps from 1-azoniabicyclo[4.1.0]heptane tosylate **2**, which was easily access from commercially available chiral aziridine (Choi et al., 2017) (Scheme 2).

Synthesis of *N*-Boc-conine **5** was initiated by the preparation of bicyclic aziridinium ion **2** (Choi et al., 2017) followed by its ring expansion with EtMgBr and CuI in 1,4-dioxane as solvent to yield the compound **3b** in 64% yield in two steps. One-pot debenzylation followed by Boc-protection furnished *N*-Boc-conine **5** in 70% yield whose Boc group would be removed by the known procedure yielding the natural product conine **6**. Analytical data for compound **5** were found in good agreement with reported value in literature (Hirai and Nagatsu, 1994; Jo et al., 1999).

**Scheme 3:**
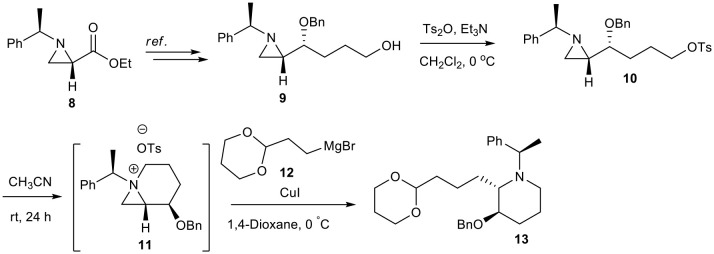
Synthesis of advance intermediate **13**.

The synthesis of (+)-epiquinamide **7** was the next task by use of our newly developed protocol with the chain extension of C2 of piperidine. Epiquinamide **7** is a quinolizidine alkaloid isolated from skin of *Epipedobates tricolor*, an Ecuadorian poisonous frog. It has been found to be highly selective toward β2 nicotinic acetylcholine receptors (nAChRs) involving in cellular signaling in both the central (CNS) and peripheral nervous systems (PNS). The importance of this receptor encourages us to utilize our strategy for its synthesis. The formal synthesis of (+)-epiquinamide **7** began with our previously prepared hydroxybutyl aziridine **9** from aziridine carboxylate **8** (Scheme 3) (Choi et al., 2017).

Tosylation of compound **9** with *p*-toluenesulphonic anhydride and triethylamine in dry CH_2_Cl_2_ gives corresponding tosylate **10** in quantitative yields. Compound **10** was kept in dry CH_3_CN under N_2_ atmosphere for 24 h to attain complete conversion to bicyclic aziridinium ion **11**, which was confirmed by NMR spectrum. Aziridine ring expansion of **11** with Gilman's reagent generated from Grignard reagent **12** and CuI gives piperidine **13** in 57% yields.

**Scheme 4:**
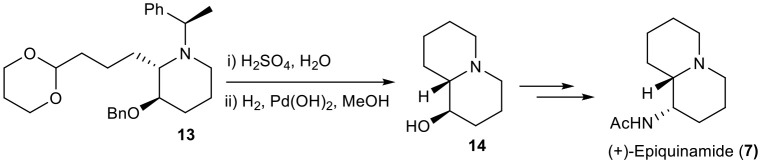
Formal synthesis of (+)-Epiquinamide (**7**).

Compound **13** was treated under acidic condition to retrieve an aldehyde from its protected acetal functionality which was subjected to one-pot reductive amination by catalytic hydrogenation under atmospheric H_2_ to get the bicyclic compound **14** in 59% yield which is able to yield epiquinamide (Scheme 4).

## Conclusion

In conclusion, we have developed a new and highly efficient method for the selective formation of 2-alkylpiperidine by the alkylative, regio- and stereospecific ring opening of highly strained bicyclic aziridinium ion with organocopper reagent. This method was applied for the synthesis of important alkaloids including conine and (+)-epiquinamide in highly efficient manner.

## Experimental Section

### Materials and Methods

Chiral aziridine-2-carboxylates are available as their menthyl ester from Sigma-Aldrich as reagents and from Imagene Co., Ltd. (http://www.imagene.co.kr/) in bulk quantities. Their corresponding ethyl esters were also available either from transesterification of menthyl ester or from Imagene. Reagents are commercially available. All commercially available reagents were used as received unless stated otherwise. Acetonitrile used to make bicyclic aziridinium ion and 1,4-dioxane used in next reactions are purified and used using calcium hydride. All reactions were carried out under an atmosphere of nitrogen in oven-dried glassware with magnetic stirrer. Reactions were monitored by thin layer chromatography (TLC) with 0.25 mm E. Merck pre-coated silica gel plates (60 F254). Visualization was accomplished with either UV light, or by immersion in solutions of ninhydrin, *p*-anisaldehyde, or phosphomolybdic acid (PMA) followed by heating on a hot plate for about 10 sec. Flash column chromatography was carried out using Intertec silica gel (Particle size: 70–230 mesh). ^1^H-NMR and ^13^C-NMR spectra were obtained using Varian unity lNOVA 400WB (400 MHz) or Bruker AVANCE III HD (400 MHz) spectrometer. Chemical shifts are reported relative to chloroform (δ = 7.26) for ^1^H NMR and chloroform (δ = 77.2) for ^13^C NMR, acetonitrile (δ = 1.94) for ^1^H NMR and acetonitrile (δ = 1.32) for ^13^C NMR, dimethyl sulfoxide (δ = 3.33) for ^1^H NMR and dimethyl sulfoxide (δ = 39.5) for ^13^C NMR. Data are reported as (br = broad, s = singlet, d = doublet, t = triplet, q = quartet, p = quintet, m = multiplet). Coupling constants are given in Hz. Ambiguous assignments were resolved on the basis of standard one dimensional proton decoupling experiments. Optical rotations were obtained using Rudolph Autopol III digital polarimeter and JASCO P-2000.Optical rotation data was reported as follows: [α]^20^ (concentration *c* = g/100 mL, solvent). High resolution mass spectra were recorded on a 4.7 Tesla IonSpec ESI-TOFMS, JEOL (JMS-700) and AB Sciex 4800 Plus MALDI TOF^TM^ (2,5-dihydroxybenzoic acid (DHB) matrix was used to prepare samples for MS. Data was obtained in the reflector positive mode with internal standards for calibration) (Supplementary Materials).

### (6*R*)-1-[(*R*)-1-Phenylethyl)-1-Azoniabicyclo[4.1.0]Heptane Tosylate (2)

*p*-Toluenesulfonic anhydride (489 mg, 1.50 mmol) was added to a stirring solution of alcohol **1** (300 mg, 1.36 mmol) and Et_3_N (0.42 mL, 3.0 mmol) in CH_2_Cl_2_ at 0°C. The resulting mixture was stirred for 45 min, warmed to RT, and stirred it for a further additional 30 min. The reaction mixture was quenched with water (15 mL), extracted with CH_2_Cl_2_ (3 × 50 mL), dried over anhydrous Na_2_SO_4_, and concentrated under vacuum to afford the crude tosylate, purified by column chromatography to give pure tosylate as a viscous liquid (486 mg, 95% yield).

Freshly prepared tosylate was stored in CD_3_CN for 24 h to allow complete conversion into azoniabicycle **2**, which was characterized by NMR spectroscopy.

^**1**^**H NMR** (400 MHz, CD_3_CN) δ 7.61 (d, *J* = 8.1 Hz, 2H), 7.63–7.48 (m, 5H), 7.15 (d, *J* = 7.8 Hz, 2H), 4.11 (q, *J* = 6.9 Hz, 1H), 3.81–3.73 (m, 1H), 3.49 (dt, *J* = 13.3, 5.3 Hz, 1H), 3.23 (dd, *J* = 9.0, 4.2 Hz, 1H), 3.15 (ddd, *J* = 13.4, 10.3, 4.9 Hz, 1H), 3.03 (dd, *J* = 7.9, 4.2 Hz, 1H), 2.33 (s, 3H), 2.12–2.04 (m, 2H), 1.72 (d, *J* = 7.0 Hz, 3H), 1.53–1.44 (m, 1H), 1.37–1.29 (m, 2H), 0.94–0.82 (m, 1H); ^**13**^**C NMR** (CD_3_CN, 101 MHz): δ 146.6, 139.6, 134.7, 131.1, 130.2, 129.5, 129.3, 126.6, 71.7, 49.7, 49.4, 43.1, 21.2, 20.8, 20.7, 15.5, 14.1.

### Typical Procedure for the Synthesis of Various Piperidine (3)

To a stirred solution of alcohol **1** (200 mg, 0.913 mmol) and Et_3_N (0.28 mL, 0.570 mmol) in CH_2_Cl_2_ (4 mL) at 0°C was added *p*-toluenesulfonic anhydride (328 mg, 1.00 mmol) and the resulting mixture was stirred for another 45 min and then warmed to rt and allowed to stir for 30 min. Reaction mixture was concentrated under vacuums and crude tosylate was dissolved in MeCN (4.0 mL) and allowed for complete conversion to azoniabicycle **2** for 24 h. Removal of MeCN solvent from tosylated azonia **2**, added dry 1,4-dioxane (5 mL) followed by CuI (1.5 eq) and Grignard reagent (3.0 eq) at 0°C. The reaction mixture was allowed to stir for appropriate time. After completion of starting material as confirmed by TLC, the reaction mixture was quenched with water (5 mL). Combined reaction mixture were filtered over Celite, followed by extracted with EtOAc (2 × 15 mL), dried over anhydrous Na_2_SO_4_, and concentrated *in vacuo* to get crude product, which was easily purified by silica gel chromatography to get pure alkylated compound **3**.

### (*S*)-2-Butyl-1-((*R*)-1-Phenylethyl)Piperidine (3a)

**[α]^20^_D_** = +51.2 (*c* = 0.7, CHCl_3_); ^**1**^**H NMR** (400 MHz, CDCl_3_) δ 7.33–7.16 (m, 5H), 4.04 (q, *J* = 6.8 Hz, 1H), 2.74 (ddd, *J* = 11.5, 5.6, 2.1 Hz, 1H), 2.41–2.32 (m, 1H), 2.25 (ddd, *J* = 16.7, 10.2, 6.8 Hz, 1H), 1.66–1.51 (m, 5H), 1.41–1.33 (m, 4H), 1.31–1.19 (m, 5H), 1.14–1.01 (m, 1H), 0.92–0.84 (t, *J* = 7.0 *Hz*, 3H); ^**13**^**C NMR** (101 MHz, CDCl_3_) δ 143.57, 128.03, 126.64, 57.51, 56.06, 44.56, 29.41, 28.27, 28.01, 25.78, 23.31, 21.88, 21.04, 14.33; HRMS-MALDI (*m/z*): calcd. For C_17_H_27_N [*M*+H]^+^ 246.1426; found:246.1422.

### (*S*)-1-((*R*)-1-Phenylethyl)-2-Propylpiperidine (3b)

**[α]^20^_D_** = +48.8 (*c* = 0.45, CHCl_3_); ^**1**^**H NMR** (400 MHz, CDCl_3_) δ 7.32–7.22 (m, 5H), 4.03 (q, *J* = 6.8 Hz, 1H), 2.78–2.67 (m, 1H), 2.44–2.35 (m, 1H), 2.32–2.22 (m, 1H), 1.66–1.41 (m, 6H), 1.40–1.33 (m, 3H), 1.31–1.19 (m, 3H), 1.11 (dddd, *J* = 20.2, 13.1, 8.3, 5.0 Hz, 1H), 0.87 (t, *J* = 7.3 Hz, 3H); ^**13**^**C NMR** (101 MHz, CDCl_3_) δ 143.71, 128.02, 126.64, 57.54, 55.77, 44.47, 30.57, 29.32, 25.73, 21.80, 21.07, 19.29, 14.68; **HRMS**-MALDI (*m/z*): calcd. For C_16_H_25_N [*M*+H]^+^ 232.1310; found:232.1319.

### (*R*)-2-Phenethyl-1-((*R*)-1-Phenylethyl)Piperidine (3c)

**[α]^20^_D_** = + 96.3 (*c* = 0.65, CHCl_3_); ^**1**^**H NMR** (400 MHz, CDCl_3_) δ 7.36–7.11 (m, 10H), 4.02 (q, *J* = 6.8 Hz, 1H), 2.71 (m, 1H), 2.61–2.50 (m, 2H), 2.44–2.31 (m, 2H), 2.01–1.80 (m, 2H), 1.61 (m, 3H), 1.51–1.38 (m, 2H), 1.36 (d, *J* = 6.8 Hz, 3H); ^**13**^**C NMR** (101 MHz, CDCl_3_) δ 143.82, 143.18, 128.45, 128.42, 128.12, 127.94, 126.71, 125.74, 57.91, 55.37, 44.29, 32.54, 30.19, 29.01, 25.46, 21.53, 21.28; **HRMS**-MALDI (*m/z*): calcd. For C_21_H_27_N [*M*+H]^+^ 294.1345; found:294.1346.

### (*S*)-2-Decyl-1-((*R*)-1-Phenylethyl)Piperidine (3d)

**[α]^20^_D_** = +68.1 (*c* = 0.1, CHCl_3_); ^**1**^**H NMR** (400 MHz, CDCl_3_) δ 7.37–7.26 (m, 5H), 4.06 (q, *J* = 6.8 Hz, 1H), 2.75 (m, 1H), 2.39 (s, 1H), 2.34–2.23 (m, 1H), 1.63–1.55 (m, 5H), 1.39 (d, *J* = 6.7 Hz, 3H), 1.31 (m, 18H), 1.13 (m, 1H), 0.94–0.86 (t, *J* = 6.8 *Hz*, 3H); ^**13**^**C NMR** (101 MHz, CDCl_3_) δ 143.57, 128.09, 126.67, 56.10, 44.61, 32.08, 30.26, 29.81, 29.52, 28.33, 25.98, 25.81, 22.85, 21.94, 21.00, 14.26; **HRMS**-MALDI (*m/z*): calcd. For C_23_H_39_N [*M*+H]^+^ 330.2336; found:330.2334.

### (*R*)-2-(But-3-En-1-Yl)-1-((*R*)-1-Phenylethyl)Piperidine (3e)

**[α]^20^_D_** = +58.8 (*c* = 1.2, CHCl_3_); ^**1**^**H NMR** (400 MHz, CDCl_3_) δ 7.32–7.19 (m, 5H), 5.87–5.70 (m, 1H), 5.04–4.85 (m, 2H), 4.04–3.95 (m, 1H), 2.77–2.64 (m, 1H), 2.51–2.42 (m, 1H), 2.32 (ddd, *J* = 15.9, 9.4, 5.9 Hz, 1H), 2.07–1.95 (m, 1H), 1.92–1.81 (m, 1H), 1.80–1.68 (m, 1H), 1.66–1.38 (m, 5H), 1.35 (d, *J* = 6.8 Hz, 3H), 1.34–1.23 (m, 2H); ^**13**^**C NMR** (101 MHz, CDCl_3_) δ 144.00, 139.46, 128.20, 128.07, 126.81, 114.30, 57.95, 55.34, 44.42, 30.53, 29.02, 27.55, 25.52, 21.68, 21.31; **HRMS**-MALDI (*m/z*): calcd. For C_17_H_25_N [*M*+H]^+^ 244.1270; found:244.1272.

### (*S*)-2-Ethyl-1-((*R*)-1-Phenylethyl)Piperidine (3f)

**[α]^20^_D_** = +63.4 (*c* = 0.62, CHCl_3_); ^**1**^**H NMR** (400 MHz, CDCl_3_) δ 7.34–7.18 (m, 5H), 4.06 (q, *J* = 6.8 Hz, 1H), 2.79–2.71 (m, 1H), 2.32–2.15 (m, 2H), 1.72–1.43 (m, 6H), 1.38 (d, *J* = 6.8 Hz, 3H), 1.27–1.13 (m, 2H), 0.79 (t, *J* = 7.5 Hz, 3H); ^**13**^**C NMR** (101 MHz, CDCl_3_) δ 143.14, 128.10, 127.99, 126.65, 57.45, 57.27, 44.73, 29.03, 25.88, 22.14, 21.33, 20.74, 10.23; **HRMS**-MALDI (*m/z*): calcd. For C_15_H_23_N [*M*+H]^+^ 218.1153; found:218.1156.

### (*R*)-2-Benzyl-1-((*R*)-1-Phenylethyl)Piperidine (3g)

**[α]^20^_D_** = −8.8 (*c* = 1.05, CHCl_3_); ^**1**^**H NMR** (400 MHz, CDCl_3_) δ 7.36 (dt, *J* = 14.9, 4.5 Hz, 4H), 7.29–7.22 (m, 1H), 7.19–7.06 (m, 3H), 6.85 (d, *J* = 7.0 Hz, 2H), 4.02 (q, *J* = 6.6 Hz, 1H), 3.04 (dd, *J* = 12.7, 3.3 Hz, 1H), 2.79–2.58 (m, 4H), 1.65–1.60 (m, 2H), 1.39 (d, *J* = 6.6 Hz, 3H), 1.36–1.29 (m, 2H), 1.28–1.22 (m, 2H); ^**13**^**C NMR** (101 MHz, CDCl_3_) δ 144.79, 141.17, 129.30, 128.44, 128.31, 127.93, 126.98, 125.70, 59.67, 57.57, 44.25, 31.71, 27.91, 26.29, 22.14, 20.51; **HRMS**-MALDI (*m/z*): calcd. For C_20_H_25_N [*M*+H]^+^ 280.1245; found:280.1240.

### (*S*)-2-Pentyl-1-((*R*)-1-Phenylethyl)Piperidine (3h)

**[α]^20^_D_** = +53.2 (*c* = 0.65, CHCl_3_); ^**1**^**H NMR** (400 MHz, CDCl_3_) δ 7.33–7.19 (m, 5H), 4.04 (q, *J* = 6.8 Hz, 1H), 2.72 (m, 1H), 2.39–2.34 (m, 1H), 2.32–2.23 (m, 1H), 1.62–1.50 (m, 4H), 1.36 (d, *J* = 6.8 Hz, 3H), 1.33–1.06 (m, 10H), 0.87 (t, *J* = 7.0 *Hz*, 3H); ^**13**^**C NMR** (101 MHz, CDCl_3_) δ 143.62, 128.06, 128.03, 126.66, 57.62, 56.09, 44.58, 32.49, 29.42, 28.25, 25.80, 25.69, 22.82, 21.89, 21.03, 14.22; **HRMS**-MALDI (*m/z*): calcd. For C_18_H_29_N [*M*+H]^+^ 260.1593; found:260.1594.

### (*R*)-2-Isobutyl-1-((*R*)-1-Phenylethyl)Piperidine (3i)

**[α]^20^_D_** = +41.2 (*c* = 1.1, CHCl_3_); ^**1**^**H NMR** (400 MHz, CDCl_3_) δ 7.36–7.17 (m, 5H), 3.97 (q, *J* = 6.7 Hz, 1H), 2.69–2.60 (m, 2H), 2.46 (m, 1H), 1.70–1.40 (m, 6H), 1.39–1.23 (m, 6H), 0.87 (d, *J* = 6.2 *Hz*, 3H), 0.69 (d, *J* = 6.1 *Hz)*; ^**13**^**C NMR** (101 MHz, CDCl_3_) δ 145.15, 128.11, 127.82, 126.61, 58.30, 52.98, 43.73, 35.98, 28.45, 25.28, 25.24, 24.19, 21.99, 21.93, 20.81; **HRMS**-MALDI (*m/z*): calcd. For C_17_H_27_N [*M*+H]^+^ 246.1476; found:246.1479.

### (*R*)-2-Isopentyl-1-((*R*)-1-Phenylethyl)Piperidine (3j)

**[α]^20^_D_** = +52.3 (*c* = 2.35, CHCl_3_); ^**1**^**H NMR** (400 MHz, CDCl_3_) δ 7.31–7.19 (m, 5H), 4.03 (q, *J* = 6.8 Hz, 1H), 2.77–2.69 (m, 1H), 2.38–2.24 (m, 2H), 1.65–1.37 (m, 7H), 1.36 (d, *J* = 6.8 Hz, 3H), 1.30–1.19 (m, 2H), 1.12 (dddd, *J* = 16.7, 11.9, 6.8, 5.2 Hz, 1H), 0.96 (tdd, *J* = 9.1, 5.0, 4.2 Hz, 1H), 0.88 (dd, *J* = 6.7, 2.7 Hz, 6H); ^**13**^**C NMR** (101 MHz, CDCl_3_) δ 143.60, 128.02, 126.64, 57.68, 56.28, 44.60, 35.21, 29.40, 28.61, 25.87, 25.81, 22.94, 22.74, 21.86, 21.01; **HRMS**-MALDI (*m/z*): calcd. For C_18_H_29_N [*M*+H]^+^ 260.1523; found:260.1526.

### (*R*)-2-(4-Chlorobenzyl)-1-((*R*)-1-Phenylethyl)Piperidine (3k)

**[α]^20^_D_** = +3.6 (*c* = 1.28, CHCl_3_); ^**1**^**H NMR** (400 MHz, CDCl_3_) δ 7.40–7.23 (m, 5H), 7.16–7.08 (m, 2H), 6.75 (d, *J* = 8.3 Hz, 2H), 3.97 (q, *J* = 6.6 Hz, 1H), 2.95 (m, 1H), 2.77–2.56 (m, 4H), 1.68–1.53 (m, 3H), 1.38 (d, *J* = 6.6 Hz, 3H), 1.28–1.13 (m, 3H); ^**13**^**C NMR** (101 MHz, CDCl_3_) δ 144.66, 139.56, 131.36, 130.49, 128.43, 128.33, 127.80, 127.00, 59.77, 57.34, 44.13, 30.85, 27.63, 26.06, 22.13, 20.28; **HRMS**-MALDI (*m/z*): calcd. For C_20_H_24_ClN [*M*+H]^+^ 313.9880; found:313.9882.

### (*R*)-2-(Cyclopentylmethyl)-1-((*R*)-1-Phenylethyl)Piperidine (3l)

**[α]^20^_D_** = +96.7 (*c* = 0.395, CHCl_3_); ^**1**^**H NMR** (400 MHz, CDCl_3_) δ 7.33–7.19 (m, 5H), 3.98 (q, *J* = 6.7 *Hz*, 1H), 2.73–2.55 (m, 2H), 2.51–2.41 (m, 1H), 1.82–1.38 (m, 16H), 1.33 (d, *J* = 6.7 Hz, 3H) 1.15–1.02 (m, 1H); ^**13**^**C NMR** (101 MHz, CDCl_3_) δ 144.98, 128.10, 127.83, 126.63, 58.37, 54.33, 43.90, 37.37, 33.99, 33.09, 32.05, 28.57, 25.39, 25.20, 25.00, 21.85, 20.96; **HRMS**-MALDI (*m/z*): calcd. For C_19_H_29_N [*M*+H]^+^ 272.0563; found:272.0562.

### (*S*)-Tert-Butyl 2-Propylpiperidine-1-Carboxylate: (+)-Boc-Coniine (5)

To a solution of compound **3b** (0.3g, 1.296 mmol) in MeOH. Pd(OH)_2_ (0.455 g, 2.24 mmol) and Boc_2_O (0.566 g, 2.59 mmol) were added. The reaction mixture was allowed to stir under hydrogenation atmospheric pressure of hydrogen for 12 h. The reaction mixture was filtered over Celite pad by using MeOH as solvent, concentrated *in vacuo* to get crude product which was purified by column chromatography to give pure product **5** (206 mg, 70 %).

**[α]^20^_D_** = +16.072 (*c* = 1.0, CHCl_3_); ^**1**^**H NMR** (400 MHz, CDCl_3_) δ 4.21 (s, 1H), 3.96 (d, *J* = 11.9 Hz, 1H), 2.74 (m, 1H), 1.65–1.54 (m, 5H), 1.45 (s, 9H), 1.43–1.20 (m, 5H), 0.92 (t, *J* = 7.3 Hz, 3H); ^**13**^**C NMR** (101 MHz, CDCl_3_) δ 155.36, 79.09, 50.32, 38.83, 32.10, 28.67, 27.59, 25.88, 19.66, 19.23, 14.24; **HRMS**-MALDI (*m/z*): calcd. For C_13_H_25_NO_2_ [*M*+Na]^+^ 250.0227; found:250.0229.

### (*R*)-4-(Benzyloxy)-4-((*S*)-1-((*R*)-1-Phenylethyl)Aziridin-2-yl)Butyl-4-Methylbenzenesulfonate (10)

*p*-Toluenesulfonic anhydride (300 mg, 0.92 mmol) was added to a stirring solution of **9** (330 mg, 1.01 mmol) and triethylamine (0.28 mL, 2.02 mmol) in CH_2_Cl_2_ at 0°C. The resulting mixture was stirred for 45 min, warmed to rt, and stirred for a further 30 min. The reaction mixture was quenched with water, extracted with CH_2_Cl_2_, dried over sodium sulfate, and concentrated under vacuum to afford the crude tosylate, which was purified by chromatography to give pure tosylate as a viscous liquid **10** (490 mg, 95% yield).

^**1**^**H NMR** (400 MHz, CD_3_CN) δ 7.79–7.64 (m, 2H), 7.42–7.39 (m, 2H), 7.36–7.17 (m, 10H), 4.53 (m, 1H), 4.31 (d, *J* = 11.7 Hz, 1H), 3.71–3.61 (m, 2H), 2.75 (td, *J* = 7.2, 3.5 Hz, 1H), 2.43 (s, 3H), 2.42–2.37 (m, 1H), 1.77 (d, *J* = 3.3 Hz, 1H), 1.51 (d, *J* = 6.2 Hz, 1H), 1.45–1.36 (m, 2H), 1.34 (d, *J* = 6.5 Hz, 3H), 1.31–1.16 (m, 2H), 1.03–0.87 (m, 1H); ^**13**^**C NMR** (101 MHz, CD_3_CN) δ 145.86, 145.52, 139.90, 133.73, 130.72, 129.79, 129.43, 128.92, 128.41, 128.30, 128.04, 127.88, 80.00, 71.72, 71.37, 70.39, 40.84, 33.76, 29.32, 24.48, 22.66, 21.42.

### (5*R*, 6*S*)-5-(Benzyloxy)-1-((*R*)-1-Phenylethyl)-1-Azoniabicyclo[4.1.0] Heptane Tosylate (11)

Freshly prepared tosylate **10** was stored in CD_3_CN for 24 h to allow complete conversion into azoniabicycle **11**, which was characterized by NMR spectroscopy.

^**1**^**H NMR** (400 MHz, CD_3_CN) δ 7.68–7.63 (m, 2H), 7.61–7.57 (m, 2H), 7.47–7.27 (m, 9H), 7.15 (dd, *J* = 8.4, 0.6 *Hz*, 2H), 4.61 (d, *J* = 11.6 *Hz*, 1H), 4.53 (d, *J* = 11.6 *Hz*, 1H), 4.31 (q, *J* = 7.0 *Hz*, 1H), 4.15–4.04 (m, 2H), 3.50–3.39 (m, 2H), 3.28–3.14 (m, 2H), 2.30 (s, 3H), 1.69 (d, *J* = 7.0 *Hz*, 3H), 1.65–1.47 (m, 2H), 1.34–1.13 (m, 2H); ^**13**^**C NMR** (101 MHz, CD_3_CN) δ 146.34, 139.76, 138.97, 134.48, 130.93, 129.90, 129.68, 129.37, 129.27, 128.58, 128.50, 126.61, 71.51, 69.58, 53.04, 48.70, 42.50, 21.85, 21.25, 15.72, 14.18.

### (2*S*,3*R*)-2-(3-(1,3-Dioxan-2-Yl)Propyl)-3-(Benzyloxy)-1-((*R*)-1-Phenylethyl)Piperidine (13)

After removal of MeCN solvent from tosylated azonia **11**, dry 1,4-dioxane (10 mL) followed by CuI (1.5 eq) and Grignard reagent **12** (3.0 eq) were added and allowed to stir at 0°C for 15 min. followed by additional 40 min. at room temperature. After completion of starting material as confirmed by TLC, the reaction mixture was quenched with water (5 mL). The Combined reaction mixture were filtered over Celite, followed by extracted with EtOAc (2 × 10 mL), dried over anhydrous Na_2_SO_4_, and concentrated *in vacuo* to get crude product, which was easily purified by silica gel chromatography to get pure alkylated compound **13** (104 mg, 57 %).

**[α]^20^_D_** = +24.012 (*c* = 0.20, CHCl_3_);**1H NMR** (400 MHz, CDCl3) δ 7.38–7.26 (m, 10H), 4.66 (d, *J* = 10.6 Hz, 1H), 4.60–4.45 (m, 2H), 4.46–4.35 (m, 1H), 4.17–4.07 (m, 1H), 3.97–3.73 (m, 3H), 3.54–3.40 (m, 1H), 2.97–2.83 (m, 2H), 2.81–2.63 (m, 1H), 2.29–2.17 (m, 1H), 2.16–1.77 (m, 4H), 1.66–1.51 (m, 2H), 1.49 (d, J = 6.8 Hz, 3H), 1.44–1.22 (m, 4H); **13C NMR** (101 MHz, CDCl3) δ 142.08, 138.70, 128.42, 128.26, 128.21, 127.93, 127.56, 127.06, 127.51, 127.48, 127.10, 102.37, 74.93, 71.02, 66.90, 60.12, 58.02, 57.13, 43.56, 27.52, 25.88, 20.49, 19.39.

### (1*R*, 9a*S*)-Octahydro-1H-Quinolizin-1-Ol (14)

To a stirred solution of **13** (100 mg, 0.236 mmol)in water (2 ml) treated with catalytic amount of sulfuric acid and stir it for 2 h, followed by quenched with NaHCO_3_. The aqueous phase was extracted with CH_2_Cl_2_. The combined organic layers were dried over anhydrous Na_2_SO_4_, concentrated *in vacuo* to get crude product, which was dissolved in MeOH (2 ml) followed by Pd(OH)_2_ (98 mg, 0.709 mmol) was added and the mixture was hydrogenated under an atmospheric pressure of hydrogen for 12 h. The reaction mixture was diluted with MeOH and filtered on a pad of Celite using MeOH as solvent. The pure product was obtained by column chromatography as viscous liquid **14** (57 mg, 59%).

**[α]^20^_D_** = −20.307 (*c* = 0.35, CHCl_3_);^**1**^**H NMR** (400 MHz, CDCl_3_) δ 4.53 (s, 1H), 3.39 (ddd, *J* = 11.1, 9.0, 4.5 *Hz*, 1H), 2.96 (d, *J* = 11.2 *Hz*, 1H), 2.84 (d, *J* = 11.4 *Hz*, 1H), 2.25–1.98 (m, 4H), 1.88–1.59 (m, 6H), 1.37–1.22 (m, 3H); ^**13**^**C NMR** (101 MHz, CDCl_3_) δ 72.76, 68.92, 56.25, 56.01, 34.12, 28.82, 25.79, 24.28, 23.45; **HRMS**-MALDI (*m/z*): calcd. For C_9_H_17_NO [*M*+H]^+^ 156.0543; found:156.0546.

## Data Availability

All datasets generated for this study are included in the manuscript and/or the Supplementary Files.

## Author Contributions

All authors listed have made a substantial, direct and intellectual contribution to the work, and approved it for publication.

### Conflict of Interest Statement

The authors declare that the research was conducted in the absence of any commercial or financial relationships that could be construed as a potential conflict of interest.
