# Supplementary material for: Alkylative Ring-Opening of Bicyclic Aziridinium Ion and Its Application for Alkaloid Synthesis
Source: Front Chem. 2019 Jun 27;7:460. doi: 10.3389/fchem.2019.00460 (PMC6610304; doi:10.3389/fchem.2019.00460)
Supplement: Supplementary file 1 [file Data_Sheet_1.doc]

**Supporting Information**

**Alkylative Ring-Opening of Bicyclic Aziridinium Ion and Its Application for Alkaloid Synthesis**

Nagendra Nath Yadav,^a^ Young-Gun Lee,^b^ Nikhil Srivastava^b^ and Hyun-Joon Ha^b^,*

^a^Department of Chemistry, North Eastern Regional Institute of Science and Technology, Nirjuli, Arunachal Pradesh-791109, India

^b^Department of Chemistry, Hankuk University of Foreign Studies, Yongin, Kyunggi-Do 17035, Korea

E-mail: [hjha@hufs.ac.kr](mailto:hjha@hufs.ac.kr)

**Table of Contents**

^1^H and ^13^C NMR spectra of **2** S2

^1^H and ^13^C NMR spectra of **3a** S3

^1^H and ^13^C NMR spectra of **3b** S4

^1^H and ^13^C NMR spectra of **3c** S5

^1^H and ^13^C NMR spectra of **3d** S6

^1^H and ^13^C NMR spectra of **3e** S7

^1^H and ^13^C NMR spectra of **3f** S8

^1^H and ^13^C NMR spectra of **3g** S9

^1^H and ^13^C NMR spectra of **3h** S10

^1^H and ^13^C NMR spectra of **3i** S11

^1^H and ^13^C NMR spectra of **3j** S12

^1^H and ^13^C NMR spectra of **3k** S13

^1^H and ^13^C NMR spectra of **3l** S14

^1^H and ^13^C NMR spectra of **5** S15

^1^H and ^13^C NMR spectra of **10** S16

^1^H and ^13^C NMR spectra of **11** S17

^1^H and ^13^C NMR spectra of **13** S18

^1^H and ^13^C NMR spectra of **14** S19

^1^H NMR of Compound **2** (400 MHz, CD_3_CN)

^13^C NMR of Compound **2** (101 MHz, CD_3_CN)

^1^H NMR Spectra of compound **3a** (400 MHz, CDCl_3_)

^13^C NMR Spectra of compound **3a** (101 MHz, CDCl_3_)

^1^H NMR Spectra of compound **3b** (400 MHz, CDCl_3_)

^13^C NMR Spectra of compound **3b** (101 MHz, CDCl_3_)

^1^H NMR Spectra of compound **3c** (400 MHz, CDCl_3_)

^13^C NMR Spectra of compound **3c** (101 MHz, CDCl_3_)

^1^H NMR Spectra of compound **3d** (400 MHz, CDCl_3_)

^13^C NMR Spectra of compound **3d** (101 MHz, CDCl_3_)

^1^H NMR Spectra of compound **3e** (400 MHz, CDCl_3_)

^13^C NMR Spectra of compound **3e** (101 MHz, CDCl_3_)

^1^H NMR Spectra of compound **3f** (400 MHz, CDCl_3_)

^13^C NMR Spectra of compound **3f** (101 MHz, CDCl_3_)

^1^H NMR Spectra of compound **3g** (400 MHz, CDCl_3_)

^13^C NMR Spectra of compound **3g** (101 MHz, CDCl_3_)

^1^H NMR Spectra of compound **3h** (400 MHz, CDCl_3_)

^13^C NMR Spectra of compound **3h** (101 MHz, CDCl_3_)

^1^H NMR Spectra of compound **3i** (400 MHz, CDCl_3_)

^13^C NMR Spectra of compound **3i** (101 MHz, CDCl_3_)

^1^H NMR Spectra of compound **3j** (400 MHz, CDCl_3_)

^13^C NMR Spectra of compound **3j** (101 MHz, CDCl_3_)

^1^H NMR Spectra of compound **3k** (400 MHz, CDCl_3_)

^13^C NMR Spectra of compound **3k** (101 MHz, CDCl_3_)

^1^H NMR Spectra of compound **3l** (400 MHz, CDCl_3_)

^13^C NMR Spectra of compound **3l** (101 MHz, CDCl_3_)

^1^H NMR Spectra of compound **5** (400 MHz, CDCl_3_)

^13^C NMR Spectra of compound **5** (101 MHz, CDCl_3_)

^1^H NMR Spectra of compound **10** (400 MHz, CD_3_CN)

^13^C NMR Spectra of compound **10** (101 MHz, CD_3_CN)

^1^H NMR Spectra of compound **11** (400 MHz, CD_3_CN)

^13^C NMR Spectra of compound **11** (101 MHz, CD_3_CN)

^1^H NMR Spectra of compound **13** (400 MHz, CDCl_3_)

^13^C NMR Spectra of compound **13** (101 MHz, CDCl_3_)

^1^H NMR Spectra of compound **14** (400 MHz, CDCl_3_)

^13^C NMR Spectra of compound **14** (101 MHz, CDCl_3_)
